# Accelerating computational fluid dynamics simulation of post-combustion carbon capture modeling with MeshGraphNets

**DOI:** 10.3389/frai.2024.1441985

**Published:** 2025-01-07

**Authors:** Bo Lei, Yucheng Fu, Jose Cadena, Amar Saini, Yeping Hu, Jie Bao, Zhijie Xu, Brenda Ng, Phan Nguyen

**Affiliations:** ^1^Lawrence Livermore National Laboratory, Livermore, CA, United States; ^2^Pacific Northwest National Laboratory, Richland, WA, United States

**Keywords:** surrogate modeling, machine learning, computational fluid dynamics, graph neural networks, carbon capture, design optimization

## Abstract

Packed columns are commonly used in post-combustion processes to capture CO_2_ emissions by providing enhanced contact area between a CO_2_-laden gas and CO_2_-absorbing solvent. To study and optimize solvent-based post-combustion carbon capture systems (CCSs), computational fluid dynamics (CFD) can be used to model the liquid–gas countercurrent flow hydrodynamics in these columns and derive key determinants of CO_2_-capture efficiency. However, the large design space of these systems hinders the application of CFD for design optimization due to its high computational cost. In contrast, data-driven modeling approaches can produce fast surrogates to study large-scale physics problems. We build our surrogates using MeshGraphNets (MGN), a graph neural network framework that efficiently learns and produces mesh-based simulations. We apply MGN to a random packed column modeled with over 160K graph nodes and a design space consisting of three key input parameters: solvent surface tension, inlet velocity, and contact angle. Our models can adapt to a wide range of these parameters and accurately predict the complex interactions within the system at rates over 1700 times faster than CFD, affirming its practicality in downstream design optimization tasks. This underscores the robustness and versatility of MGN in modeling complex fluid dynamics for large-scale CCS analyses.

## 1 Introduction

Carbon capture systems (CCSs) play a crucial role in mitigating greenhouse gas emissions from fossil fuel-based power plants and other industrial processes (Edenhofer, 2015; Koytsoumpa et al., 2018; Chao et al., 2021). Solvent-based post-combustion approach is a widely adopted technology in which carbon dioxide (CO_2_) is captured through an absorption process involving interactions between a liquid solvent and the flue gas inside a packed column (Yeh et al., 2001; Wang et al., 2017). The design and optimization of these CO_2_-capture columns is a foundational challenge, as their capture efficiency critically depends on maximizing the gas-solvent interfacial area (IA) to enhance the CO_2_ absorption reaction (Singh et al., 2017; Song et al., 2018; Fu et al., 2022).

Modeling the hydrodynamics, heat, and mass transfer in a packed column typically relies on computational fluid dynamics (CFD) simulations to numerically solve the partial differential equations (PDEs) that govern the underlying physical processes (Fu et al., 2020, 2022). While CFD methods can provide physically accurate results, they are computationally expensive to apply, especially when evaluating numerous column configurations during the design optimization process. Hence, generating CFD simulations is a bottleneck in the design optimization of a CO_2_-capture column.

Recent advances in machine learning and deep learning techniques have shown potential in accelerating traditional numerical simulations (Raissi et al., 2019; Kim et al., 2019; Brunton et al., 2020; Kochkov et al., 2021; Pfaff et al., 2020; Karniadakis et al., 2021; Lino et al., 2023; Janny et al., 2023). By leveraging data-driven models trained on existing simulations, it becomes possible to develop surrogate models that can quickly approximate the complex fluid dynamics and derive key CO_2_-capture efficiency measures, such as IA. By replacing CFD with these surrogates, this approach can substantially reduce the computational burden associated with the design optimization of CO_2_-capture columns, enabling a more thorough and rapid exploration of the configuration space and facilitating the discovery of high-performance designs.

Previously, Bartoldson et al. (2022) introduced a latent space simulator that built upon Deep Fluids (Kim et al., 2019) and leveraged autoencoders and latent space models to learn the temporal evolution of fluid flow. When trained on simulations of a CO_2_-capturing solvent flowing in a random packed column (Fu et al., 2020), their optimized surrogates achieved 4,000 × speedup with 4% relative error in predicting IA for unseen inlet velocity conditions. However, a major limitation of this model is its inability to generalize well to new packing configurations, which required retraining a separate model for each configuration.

Graph neural networks (GNNs) have emerged as a promising direction for learning mesh-based simulations directly from mesh data (Belbute-Peres et al., 2020; Pfaff et al., 2020; Lino et al., 2021; Brandstetter et al., 2022; Allen et al., 2022; Fortunato et al., 2022). GNNs operate on graphs, making them well-suited for modeling the interactions between nodes (representing mesh elements) and edges (representing connectivity) in non-uniform meshes that are typically used in CFD. Pfaff et al. (2020) introduced MeshGraphNets (MGN), a GNN framework that learns to simulate mesh dynamics accurately and generalize well across different mesh topologies. Building upon this, Bartoldson et al. (2023) proposed SCALES2, a set of scientific computing-based enhancements that can scale training to meshes with millions of nodes. They applied their methods to 2D and 3D fluid flow simulations on multiple random packing configurations of a CO_2_-capture column and demonstrated that their enhanced MGN models can scale to large, complex domains and transfer to unseen packed columns while still achieving low prediction errors.

However, the aforementioned studies only considered one design parameter (liquid inlet velocity) as a variable input (Bartoldson et al., 2022, 2023), whereas the CO_2_-capture efficiency of a column can be affected by multiple design and operating conditions (Fu et al., 2022). In this study, we build on the work by Bartoldson et al. (2023) and consider an expanded dataset with multi-parametric inputs. We introduce contact angle and surface tension as additional packing and solvent-related design parameters to impose additional physical diversity onto the fluid simulations and broaden the design optimization space of operating conditions within a packed column. Different combinations of the liquid inlet velocity, contact angle, and surface tension can produce drastic differences in the fluid flow, making surrogate modeling of this higher-dimensional dataset an extremely challenging task. We demonstrate that MGN can be successfully trained on these datasets to capture these differences while still producing fast, accurate surrogates that can be incorporated into large-scale, multi-dimensional design optimization problems.

## 2 Method

### 2.1 Dataset

**Figure 1:**
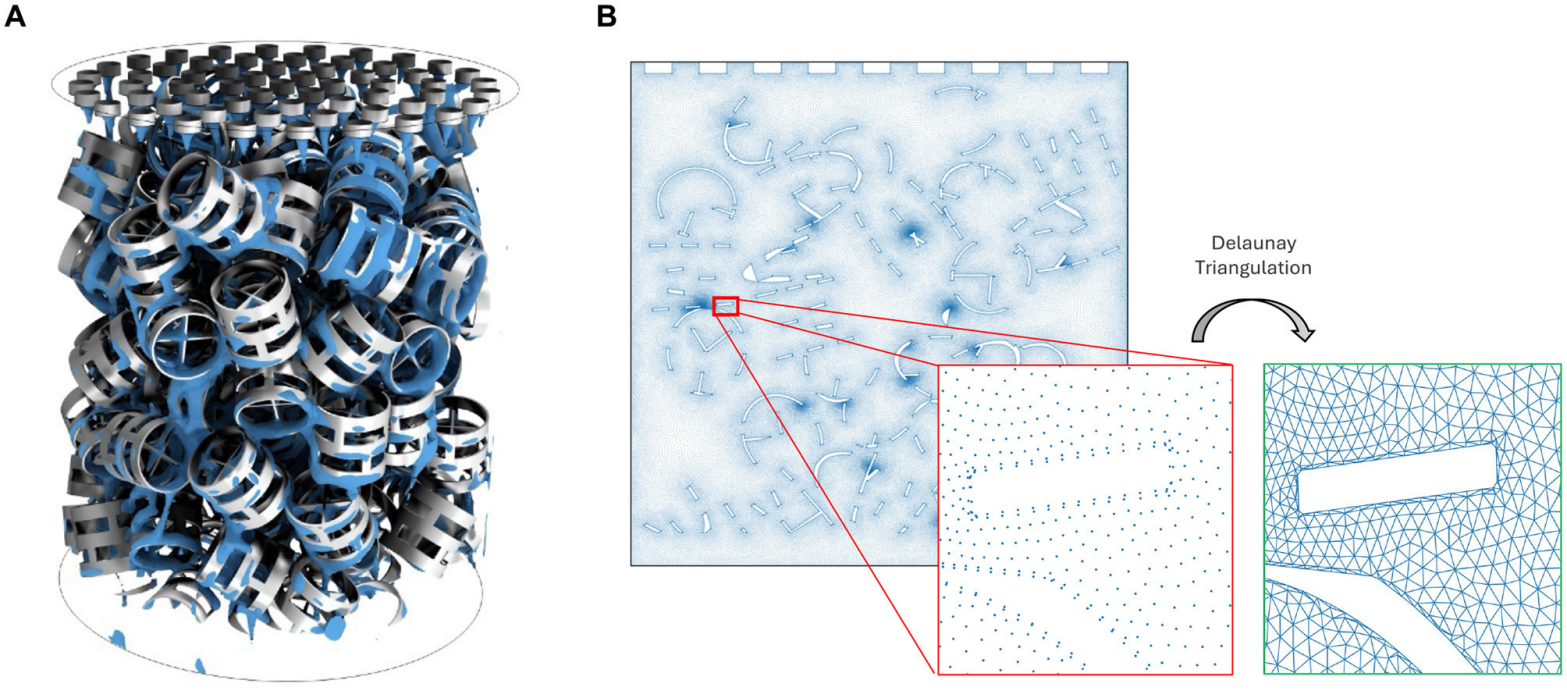
**(A)** Visualization of a 3D CO_2_-capture column packed with pall rings. **(B)** 2D cross section of the 3D column. Mesh edges are created by Delaunay triangulation.

Our dataset consists of CFD simulations that model the liquid–gas countercurrent flow hydrodynamics inside a CO_2_-capture column (see Figure 1). The main objective for this dataset is training models that can accurately predict the liquid volume fraction and momentum at each position and timestep of the computational domain. In each simulation, a CO_2_-capturing solvent flows from top to bottom across pall rings that are randomly packed in the column, which aim to spread the solvent across a wide surface area on the rings to maximize the liquid-gas contact area; example snapshots of these simulations may be found in **Figure 5** and Supplementary Figures S2–S4.

We use the same setup described in Fu et al. (2020) and Bartoldson et al. (2023): Eulerian simulations of the counter-current solvent and gas flow are generated by numerically solving the continuity and momentum equations with STAR-CCM+ and modeling the multiphase fluid separation with the volume-of-fluid method. The continuity equation is given by


(1) $$\begin{array}{l} \frac{\partial \rho }{\partial t}+\nabla \cdot \left(\rho \text{u}\right)=0, \end{array}$$

where ρ is the density and **u** is the velocity field. The momentum conservation equation is given by


(2) $$\begin{array}{l} \frac{\partial \left(\rho \text{u}\right)}{\partial t}+\nabla \cdot \left(\rho \text{u}\text{u}\right)=-\nabla p+\mu \nabla^{2}\text{u}+\rho \text{g}+{\text{F}}_{\sigma }, \end{array}$$

where *p* is the pressure, μ is the viscosity, **g** is gravity, and **F**_σ_ is the surface tension force at the gas-liquid interface. The density and viscosity are computed as a volume fraction average of the liquid (α) and gas phase (1−α). The interfacial surface tension force **F**_σ_ is computed as


(3) $$\begin{array}{l} {\text{F}}_{\sigma }=\sigma \kappa \nabla \alpha ,\;\kappa =-\nabla \cdot \frac{\nabla \alpha }{\left|\nabla \alpha \right|}, \end{array}$$

where σ is the surface tension coefficient and κ is the local surface mean curvature. On the packing wall region, $\frac{\nabla \alpha }{\left|\nabla \alpha \right|}$ is computed as *n*_*w*_cosθ+*t*_*w*_sinθ, where *n*_*w*_ and *t*_*w*_ are the unit normal and tangential vectors of the wall surface, respectively. The contact angle θ is the angle between the gas–liquid interface and the solid surface. Finally, the evolution of the liquid volume fraction α is governed by the transport equation


(4) $$\begin{array}{l} \frac{\partial \alpha }{\partial t}+\nabla \cdot \left(\text{u}\alpha \right)=0. \end{array}$$

We generate 2D simulations by taking a cross-section of the 3D column (Figure 1A) and solving the governing equations on that cross-section. STAR-CCM+ uses a timestep size of 0.001s to solve the equations, but the data is saved at intervals of 0.01s. Each simulation spans 500 timesteps; the number of timesteps is chosen such that each simulation reaches a pseudo-steady-state (stable behavior) by the end of the simulation. The 2D column is represented by a computational mesh that contains 164,715 nodes, and each node corresponds to a physical location in the domain and contains measurements of its position, pressure, velocity, liquid volume fraction, and momentum per unit volume. Momentum per unit volume, or mass flow rate per unit area, is computed as


(5) $$\begin{array}{l} \text{m}=\left[\alpha \rho_{L}+\left(1-\alpha \right)\rho_{G}\right]\text{u}, \end{array}$$

where $\rho_{L}=1010{\text{kg/m}}^{3}$ and $\rho_{G}=1.18415{\text{kg/m}}^{3}$ are the liquid and gas densities, respectively. We construct edges using Delaunay triangulation. The 2D domain with mesh edges is shown in Figure 1B.

A key aspect of this dataset is the variation of three input design parameters: liquid inlet velocity **v**_inlet_, contact angle θ, and surface tension σ. Unlike previous works in which only **v**_inlet_ was varied (Bartoldson et al., 2022, 2023), the inclusion of θ and σ introduces additional complexities in training accurate surrogate models. These parameters can drastically influence the physical behavior of the solvent and the interactions between the liquid, gas, and packing structures within a CO_2_-capture column; we describe these differences in detail in Section 3.3.2. Consequently, the surrogate models must learn to adapt to and predict a larger variety of fluid dynamics behaviors in order to make accurate predictions across a wider parameter space. In total, our dataset contains 150 2D simulations, achieved by varying the contact angle, surface tension, and inlet velocity across 5, 5, and 6 discrete values, respectively. The exact values are displayed in Table 1.

**Table 1:** Discrete values for surface tension (σ), contact angle (θ), and liquid inlet velocity (**v**_inlet_).

σ (N/m)	0.01, 0.03, 0.05, 0.07, 0.09
θ (°)	10, 30, 50, 70, 90
**v**_inlet_ (m/s)	0.002, 0.00326, 0.00531, 0.00864, 0.0141, 0.0218

The dataset consists of 150 unique combinations of these design parameters.

### 2.2 MeshGraphNets

MeshGraphNets (MGN) (Pfaff et al., 2020) is a message-passing GNN that uses an encode-process-decode architecture (Battaglia et al., 2018; Sanchez-Gonzalez et al., 2020) to learn from mesh-based simulations. To make a prediction of a system at a following timestep, the encoder transforms the input mesh data at the current timestep into a graph and embeds its nodes and edges into a high-dimensional feature space. The processor then applies message-passing to summarize the physics dependencies between neighboring nodes. By applying a sequence of *k* message-passing steps, the processor forces each node and edge to summarize information about its *k*-hop neighborhood to update its embedding. The decoder then converts these latent features into node outputs that can be used to update the state of the system. Additional details about the MGN algorithm may be found in Pfaff et al. (2020).

#### 2.2.1 Modeling and training details

Our goal is to train MGN models that can accurately predict the evolution of the liquid volume fraction and momentum per unit volume throughout a random packed column. To train on our 2D simulations, for each node *i*, we include the physical location **x**_*i*_, momentum per unit volume **m**_*i*_, liquid volume fraction α_*i*_, node type **n**_*i*_, contact angle θ, and surface tension σ as input node features. Node types are one-hot encoded features that contain information about the space or boundary that is present at a node; details about the node types can be found in Supplementary Figure S1. As design inputs, the contact angle θ and surface tension σ are normalized and included in all the node input features. We normalize θ from (0°, 90°) to (−1, 1) and σ from (0, 0.1) to (−1, 1) using linear scaling. For liquid inlet velocity **v**_inlet_, we incorporate it as a design input by setting and fixing the corresponding momentum values at nodes that have a “liquid inlet” node type. Based on the default MGN settings in Pfaff et al. (2020), we use the same set of edge features (distance and relative displacement vector), 15 message-passing steps, and a hidden dimension of 128 to encode nodes and edges. Ablation studies on the number of message-passing steps and the hidden dimension size were also performed; see Supplementary Figures S6, S7.

We train MGN to predict the change in liquid volume fraction and momentum per unit volume between consecutive timesteps. Specifically, if *G*_*t*_ represents the input graph and state of the system and **y**_*t*_ represents the target state variables at time *t*, then we train MGN(*G*_*t*_) to predict Δ**y**_*t*_ = **y**_*t*+1_−**y**_*t*_. We then predict the next state as ${\hat{\text{y}}}_{t+1}={\text{y}}_{t}+\text{MGN}\left(G_{t}\right)$. Given an initial graph *G*_0_, we can generate a full simulation rollout prediction by iteratively applying the MGN model and updating the graph with its predictions.

We use 32 NVIDIA V100 16 GB GPUs and an Adam optimizer with an exponentially decaying learning rate from 1e-3 to 1e-7 over 4 million steps to train MGN models. Since we cannot fit our large 165K-node graph on a single GPU during training, we apply domain decomposition and patch training to train on smaller subgraphs in a manner that would be equivalent to training on the whole graph (Bartoldson et al., 2023). We partition the graph into a 3 × 4 grid and add “ghost” nodes to each patch so that each node that contributes to a gradient update has access to its correct 15-hop neighborhood, and we randomly sample patches across all the train simulations and timesteps to perform gradient updates. To evaluate performance for unseen input parameter configurations, we partition our dataset into 120 train and 30 test simulations using Latin hypercube sampling to ensure sufficient coverage of the design space in the test dataset. Supplementary Figure S9 provides the design parameter details for each train and test simulation.

## 3 Results

### 3.1 Evaluation metrics

We evaluate the performance of our trained models in several ways. First, we compute the root-mean-square error (RMSE) of the next-step predictions of liquid volume fraction, denoted as RMSE_VF − 1_. Since we generate a predicted rollout by repeatedly feeding the next-step predictions back into the MGN, these errors may accumulate over the course of a simulation. Therefore, we also compute the RMSE at the end of a predicted simulation (at timestep 500), denoted as RMSE_VF − 500_. Since our main purpose of training surrogate models is rapid evaluation of various parameters to maximize CO_2_-capture efficiency in a design optimization pipeline, we also compute the relative error of the steady-state interfacial area (IA). We compute IA as the total arc length of contours where the liquid volume fraction is equal to 0.5, and we compute steady-state IA as the average IA of the last 20 timesteps of a simulation. The equations of RMSE_VF − 1_, RMSE_VF − 500_, and relative IA error can be found in Section 1 of the supplementary text. Table 2 summarizes the RMSE_VF − 1_, RMSE_VF − 500_, and relative IA errors of the train and test datasets.

**Table 2:** Average one-step error RMSE_VF−1_, final-step error RMSE_VF−500_ and relative IA error for train and test simulations.

	**RMSE_VF − 1_**	**RMSE_VF − 500_**	**Relative IA error**
Train	0.029	0.310	7.6%
Test	0.030	0.313	9.2%

### 3.2 One-step prediction

Since the MGN model is trained using the one-step difference Δ**y**_*t*_ = **y**_*t*+1_−**y**_*t*_, the one-step error RMSE_VF − 1_ directly reflects its training performance. The average RMSE_VF − 1_ for the train and test datasets are 0.029 and 0.030, respectively, so the MGN model performs consistently well in one-step prediction across both train and test datasets. The model also does not overfit to the train dataset.

**Figure 2:**
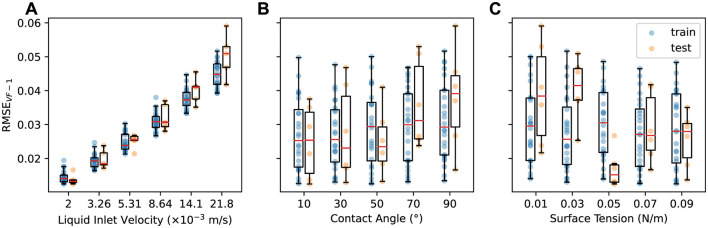
One-step error in liquid volume fraction RMSE_VF−1_ vs. **(A)** liquid inlet velocity, **(B)** contact angle, and **(C)** surface tension for the training (blue) and test (orange) datasets.

To analyze the influence of design parameters on one-step prediction, we group the results based on the values of each parameter, as shown in Figure 2. Figure 2A shows a strong correlation between the prediction error and liquid inlet velocity **v**_inlet_, with RMSE_VF − 1_ increasing as **v**_inlet_ increases in both train and test datasets. Since MGN predicts Δ**y**_*t*_ = **y**_*t*+1_−**y**_*t*_, the distribution of Δ**y**_*t*_ values can influence the resulting RMSE_VF − 1_. Faster **v**_inlet_ generally results in larger Δ**y**_*t*_ values, and as the liquid travels through column, the interactions with packings can cause the variance in velocities to increase. This can make Δ**y**_*t*_ harder to predict and ultimately lead to higher one-step errors. Additionally, while the train and test results closely align at lower **v**_inlet_, a gap in their errors emerges as **v**_inlet_ increases, suggesting that the model generalizes better at lower **v**_inlet_ while its performance declines slightly at higher **v**_inlet_. This may be due to increased complexities in interactions between fluid and packing structures at higher **v**_inlet_ (Fu et al., 2020).

Figures 2B, C show results grouped by contact angle θ and surface tension σ values, respectively. For θ, there is a slight increase in RMSE_VF − 1_ as θ rises from 10° to 90° for both train and test datasets. A large gap emerges between the train and test results when θ is 90°. For σ, the train results show consistent RMSE_VF − 1_ across all σ levels, while the test results are generally lowest at σ = 0.05, with lower σ tending to produce higher errors. Overall the one-step errors are more robust to changes in the contact angle and surface tension than to changes in liquid inlet velocity, indicating that the MGN model can make consistently accurate one-step predictions across a broad range of contact angle and surface tension values.

**Figure 3:**
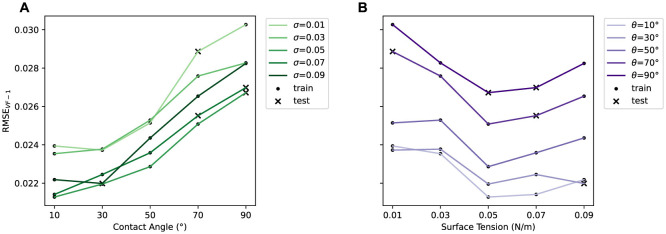
One-step error in liquid volume fraction RMSE_VF−1_ vs. **(A)** contact angle θ, and **(B)** surface tension σ for simulations with **v**_inlet_ = 0.00531. Lines connect data points with the same σ and θ, respectively. Train and test data are shown in different markers.

The errors shown in the aggregated plots in Figures 2B, C are heavily obscured by the inlet velocity behavior and do not fully capture the fine-grained relationships of the contact angle θ and surface tension σ with the one-step errors. To investigate further, we focus on a subset of the data with a fixed **v**_inlet_ of 0.00531. Figure 3 shows RMSE_VF − 1_ for this subset, with each line connecting data points sharing the same θ or σ. Consistent with the trends observed in Figure 2B, in Figure 3A, we see that RMSE_VF − 1_ increases as θ increases for all σ; a higher contact angle increases the hydrophobicity against the packing structure, leading to a larger Δ**y**_*t*_ and consequently higher prediction errors. In Figure 3B, the lowest RMSE_VF − 1_ is consistently observed at a surface tension of 0.05, regardless of the contact angle. Lower surface tension also tends to produces higher prediction errors; smaller surface tension values tends to result in the formation of smaller droplets that have higher momentum, which then amplifies Δ**y**_*t*_.

In summary, the one-step error analysis shows that the MGN model generalizes well to unseen design parameter combinations. In addition, the liquid inlet velocity has the most significant impact on the one-step error, but this error is also robust to contact angle and surface tension. Therefore, the MGN model can make consistently accurate one-step predictions across different contact angle and surface tension values.

### 3.3 Rollout prediction

While we trained MGN to minimize the one-step error predictions, these errors may accumulate over the course of a rollout. In addition, the dynamics of the rollout that are most relevant to many CO_2_-capture efficiency metrics occur when the system achieves pseudo-steady-state or stable behaviors. We now consider the errors in the final timestep predictions and IA calculations.

Once trained, we can apply the MGN model repetitively to generate predicted rollouts. We use RMSE_VF − 500_ to measure its prediction performance on the liquid volume fraction for the last time frame (*t* = 500). We also visualize the predicted rollouts and qualitatively evaluate some example cases. Lastly, we perform a computational efficiency analysis by comparing the MGN and CFD generation runtimes.

#### 3.3.1 Last-step error analysis

The MGN model achieves average RMSE_VF − 500_ values of 0.310 and 0.313 for the train and test datasets, respectively. The small difference in these values highlight the strong generalization capabilities of MGN for extended rollouts.

**Figure 4:**
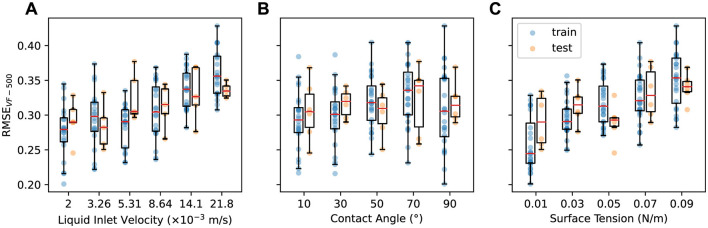
Last-step error in liquid volume fraction RMSE_VF−500_ vs. **(A)** liquid inlet velocity, **(B)** contact angle, and **(C)** surface tension for train (blue) and test (orange) datasets.

Figure 4 shows RMSE_VF − 500_ aggregated based on the design parameter values. In Figure 4A, we observe that RMSE_VF − 500_ increases with higher **v**_inlet_, consistent with the trends noted in the one-step predictions (Figure 2A). However, the correlation in this longer-term prediction is not as pronounced as in the one-step results, since errors can accumulate in a complicated manner over the course of the predicted rollout.

For contact angle θ (Figure 4B), RMSE_VF − 500_ shows a slight increase at θ = 70° for both train and test datasets. Apart from this instance, RMSE_VF − 500_ does not exhibit consistent patterns across other θ values, suggesting that the model's predictive performance is generally robust to variations in θ. In contrast, the response to variations in surface tension σ (Figure 4C) reveals a different pattern: RMSE_VF − 500_ increases with increasing σ, indicating a strong sensitivity of the accumulated error to this parameter. Notably, the model shows a greater disparity in performance between the train and test datasets at smaller σ values (0.01 and 0.03), so generalization for extended rollout predictions under these conditions needs to be improved.

In summary, our analysis demonstrates that the last-step error is particularly sensitive to changes in liquid inlet velocity and surface tension. This finding underscores the need for careful consideration of these parameters during the design optimization process to ensure robust model performance.

#### 3.3.2 Rollout visualizations

**Figure 5:**
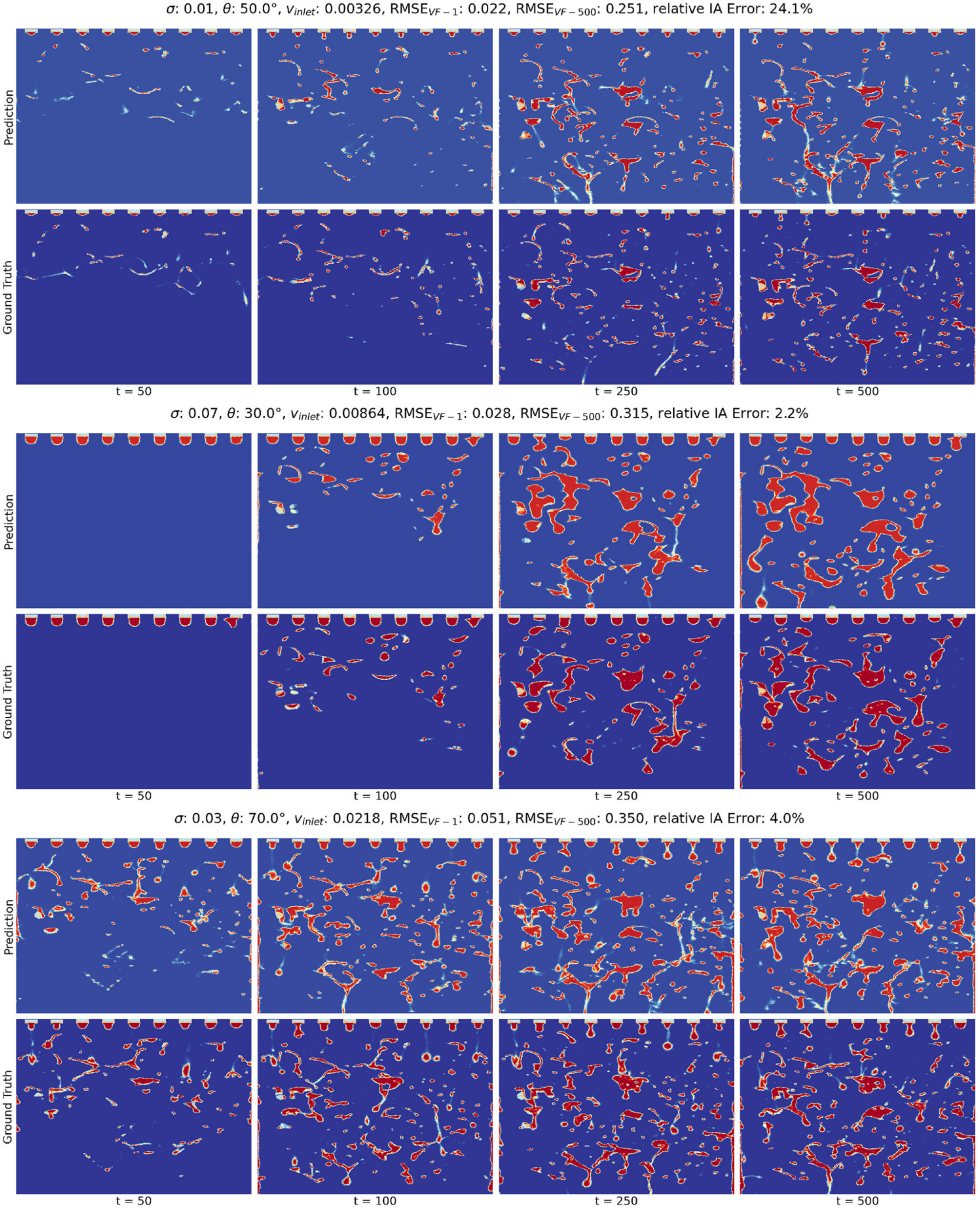
Predicted and CFD-generated rollouts of the volume fraction for 3 selected test simulations. In each subplot, the **top** and **bottom** rows correspond to the predicted and ground truth rollouts, respectively. From left to right, the timesteps are 50, 100, 250, and 500. The first case represents a slow liquid flow with small droplets and medium wettability. The second case represents a slow liquid flow with large droplets that retain on the packing structure. The third case represents a fast liquid flow with small droplets that slide through the packing structure.

Figure 5 shows the predicted evolution of the liquid volume fraction at select timepoints for three combinations of the design parameters, alongside their respective ground truth simulations. The first case features a low **v**_inlet_, medium θ, and low σ, representing a slow liquid flow with small droplets and medium wettability. The second case features a low **v**_inlet_, low θ, and high σ, representing a slow liquid flow with large droplets that retain on the packing structure. The third case features a high **v**_inlet_, high θ, and low σ, representing a fast liquid flow with small droplets that readily slide through the packing structure. Additional snapshots at other combinations of **v**_inlet_, θ, and σ are shown in Supplementary Figures S2–S4.

The ground truth rollouts illustrate the varying behaviors in the fluid dynamics across the different parameter combinations, highlighting the pronounced impact of the design parameters on the fluid behaviors. For each design parameter, we make the following observations:

- In Supplementary Figure S2, we find that liquid inlet velocity **v**_inlet_ has a direct influence on the speed and volume of liquid flow. Higher **v**_inlet_ is correlated with faster evolution of the liquid volume fraction due to the increased speed and volume.
- The contact angle θ governs the wettability of the packing structure (Singh et al., 2020). From Supplementary Figure S3, we observe that a low value of θ results in high liquid spreading over the packing surfaces, leading to higher liquid volume fraction. As θ increases, hydrophobicity increases, which causes the liquid to flow down across the packings more quickly.
- Surface tension σ exhibits a synergistic effect with the contact angle on the wettability of the packing surface (Fu et al., 2022). Additionally, it highly impacts the size and shape of the liquid droplets emerging from the inlet. As shown in Supplementary Figure S4, higher surface tension values result in larger droplets and larger volume of fluid flow, which drastically change the volume fraction profiles.

Despite the high complexity of the system, our MGN model can generate plausible predictions of the liquid volume fraction rollouts. Visually, the snapshots of the ground truth and predicted rollouts appear to be very similar and corroborate our analysis of RMSE_VF − 500_. Combined with the analysis of RMSE_VF − 500_ and RMSE_VF − 1_, these snapshots demonstrate our model's ability to adapt to different design parameters and predict a wide range of interactions between fluid and packing structures.

#### 3.3.3 Speedup over CFD

The primary advantage of the MGN model lies in its massive acceleration over the traditional numerical approaches that were used to generate the simulation datasets. To measure this performance, we calculate the relative speedup by comparing the wall-clock time required to generate one of our CFD simulations in STAR-CCM+ (60 h) to the time taken by our surrogate model for a complete rollout over the same number of timesteps. The computation time and speedup results are listed in Table 3.

**Table 3:** Acceleration performance of the MGN model over CFD simulations.

**Configuration**	**# GPU(s)**	**AMP**	**Time(s)**	**Average speedup over CFD (×)**
Full domain	1	×	357.6 ± 1.5	604
Full domain	1	✓	232.8 ± 0.7	928
Patch 2 × 2	4	×	161.5 ± 0.3	1,337
Patch 2 × 2	4	✓	121.8 ± 0.4	1,773

Computational time for a single rollout (500 timesteps) is shown. Standard deviations are computed across 10 runs. The speedup is computed using a CFD reference of 60 h.

Using a single GPU, the MGN model achieves over 500 × average speedup on the full domain graph. Domain decomposition and parallel computing with multiple GPUs can further accelerate the inference speed of the MGN model (Bartoldson et al., 2023). After applying a 2 × 2 patch decomposition with rollout distributed on 4 GPUs and automatic mixed precision (AMP) (Micikevicius et al., 2017), the MGN model reaches an average speedup of 1,773. We note that that the speedup can be further improved with additional parallelization, quantization-aware training (Jacob et al., 2018), and other efficient inference techniques.

### 3.4 IA analysis

Since the final timesteps of each simulation are used to compute the steady-state IA, the accuracy at the last timesteps may also be indicative of the IA error. We now consider the relative IA error by using our predicted rollouts to compute the IA and averaging over the last 20 timesteps of each simulation.

**Figure 6:**
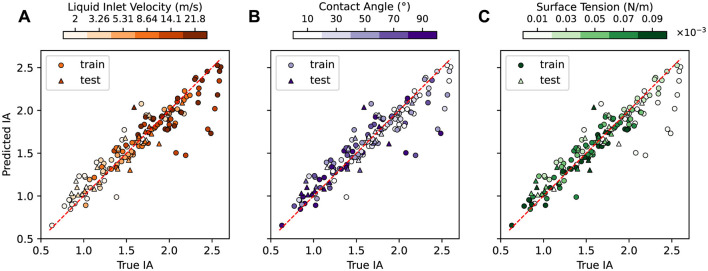
Predicted vs. true IA for the train and test simulations. The same predictions are shown in each panel but are colored by **(A)** liquid inlet velocity, **(B)** contact angle, and **(C)** surface tension of the corresponding simulation. The relative IA error for the train and test sets is 7.6% and 9.2%, respectively.

**Figure 7:**
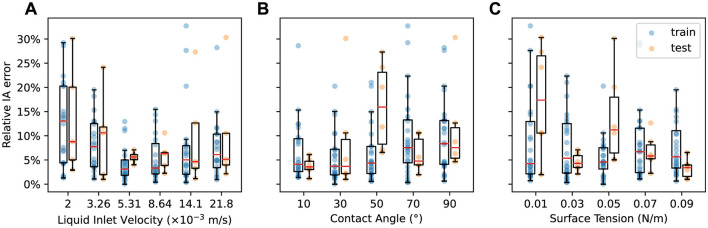
Relative IA error vs. **(A)** liquid inlet velocity, **(B)** contact angle, and **(C)** surface tension for the train (blue) and test (orange) datasets.

Figure 6 compares the predicted and true IA values for each of the 150 simulations in our dataset, with train and test data points denoted by different markers. In each subplot, points are colored by Figure 6A liquid inlet velocity **v**_inlet_, Figure 6B contact angle θ, and Figure 6C surface tension σ value of the corresponding simulation. Figure 7 shows the relative IA error aggregated by the values of each design parameter. Detailed IA results for the test simulations can be found in Supplementary Table S1.

In general, the error between the predicted and true IA values is low across the parameter space. The MGN model achieves an average relative IA error of 7.6% and 9.2% for the train and test datasets, respectively. The low average errors and small gap between the train and test results demonstrate MGN's ability to sufficiently learn the underlying physics of the countercurrent flow to accurately predict the simulations across a wide range of design parameters. However, the MGN does exhibit some differences in IA prediction quality in certain parameter ranges.

Figure 7A shows the relationship between relative IA error and liquid inlet velocity **v**_inlet_. Both train and test results exhibit a similar trend: relative IA error decreases initially with increasing **v**_inlet_, achieves the lowest values when **v**_inlet_ is in the medium range (0.00531, 0.00864), but then increases beyond the intermediate values of **v**_inlet_. The worst performance occurs at the extreme values of **v**_inlet_. Supplementary Figure S5 shows two test simulations for such cases with relative IA error over 30%. The first case features the lowest **v**_inlet_. The model makes good predictions of the volume fraction until near the end of the simulation, when some of the volume fraction values at the lower left region are over-predicted. The second case features the highest **v**_inlet_, lowest σ and highest θ, representing the fastest liquid flow in all of the simulations. The model fails to predict volume fraction accurately from the very beginning, possibly due to the extremely fast evolution of momentum and volume fraction of the liquid. Since the IA calculation is based on the contour surfaces of these volume fraction values, this subsequently leads to large errors in IA.

The influence of contact angle θ and surface tension σ on the IA prediction performance is shown in Figures 7B, C, respectively. In general, the relative IA error for the train dataset tends to be low and similarly distributed for the smaller θ (10° to 50°). A slightly higher average error is found for large θ (70° and 90°). Test results exhibit similar trend, except for the high error at θ = 50°. For σ, the relative IA error for the train dataset tends to be similarly distributed at most of the σ values. The test results again show some high errors and large variations for σ values of 0.01 and 0.05, partially due to the influence of other two design parameters. Overall, the MGN's IA prediction error is mostly sensitive to liquid inlet velocity, but generally robust to contact angle and surface tension, consistent with our earlier analyses.

We note that while we expect the rollout error to impact the IA error, high IA errors can still occur as a result of the MGN having difficulties predicting the simulation within certain regions of the column or due to complexities of the fluid behavior at certain parameter settings. For example, in the first case in Figure 5, the predicted rollout closely resembles the ground truth for the majority of the volume fraction predictions and achieves a low RMSE_VF − 500_ at 0.251, but the predicted IA has a high relative error of 24.1%. The high error can be ascribed to the suboptimal predictions at the lower left of the simulation domain, where over-prediction happens from timestep 250. This observation highlights that the computation of IA may be sensitive to specific regions within the simulation domain, and a high relative IA error does not necessarily imply inaccurate predictions throughout the entire domain.

Overall, the MGN model successfully predicts the IA, achieving an average relative IA error of 9.2% on the test dataset. This error is sensitive to the liquid inlet velocity, but the model performs the best when intermediate velocity values are used. The model also has lower IA errors for low to medium contact angle values. Finally, the error is generally robust to contact angle and surface tension differences, but certain regions of the column as well as certain combinations of design parameters can make IA harder to predict.

Given the model's demonstrated accuracy in predicting IA across the design parameter space, it can be used in various design optimization applications to screen and assess candidate column designs. In addition, by understanding the parameters that affect IA most, we can refine our design optimization strategies to target specific improvements. We now demonstrate how we utilize the surrogate model to optimize design configurations to maximize CO_2_-capture efficiency.

### 3.5 Application to design optimization

We use our surrogate model to demonstrate a design optimization task where the goal is to find a configuration of liquid inlet velocity **v**_inlet_, contact angle θ, and surface tension σ that maximizes the IA. Without additional restrictions, IA generally increases as inlet velocity increases, contact angle decreases, and surface tension decreases. In practice, however, designs for carbon capture systems will be subject to different types of constraints (e.g., financial and structural), and the interactions between different design parameters may be difficult to characterize. In addition, the traditional CFD approach represents a bottleneck in the design optimization pipeline, making it difficult to assess more than a few design parameter configurations in a reasonable amount of time. Here, as a mode of example, we consider a simple L1 penalty term to discourage designs with extreme values in the configuration.

More concretely, we write IA as a function of the aforementioned design variables:


$$\text{IA}=f\left({\text{v}}_{\text{inlet}},\theta ,\sigma \right),$$

where *f* is a function that runs 500 timesteps of rollout using our surrogate model with the given configuration. Then, we solve the following optimization problem:


(6) $$\begin{array}{l} \underset{v_{\text{inlet}},\;\theta ,\;\sigma }{\max}\;f(v_{\text{inlet}},\;\theta ,\;\sigma )-\lambda (|v_{\text{inlet}}|+|\theta |+|\sigma |)\\ \text{ s}.\text{t}.\;v_{\text{inlet}}\in [0.001,0.03]\\ \;\theta \in (-1,1)\\ \;\sigma \in (-1,1) \end{array}$$

where λ is a regularization parameter, and θ and σ have been normalized as described in Section 2.2. We set λ to 0 (unconstrained), 1, 5, and 10.

We perform optimization using the *minimize* procedure in the scipy module for Python, allowing the procedure to run for 50 simulations. We show the results of the optimization in Table 4. As expected, in the unconstrained case, IA is maximized by a high **v**_inlet_, low θ, and low σ. As the penalty parameter increases, this solution at the extreme of the parameter space is no longer ideal. Instead, the objective function is maximized by a configuration in the middle of the (normalized) range for all parameters.

**Table 4:** Designs that maximize interfacial area subject to different cost functions.

**λ**	**σ (N/m)**	**θ(°)**	**v_inlet_ (m/s)**	**Objective function**
0	0.01	11.25	0.03	3.077
1	0.0255	45.0	0.03	2.452
5	0.05	45.0	0.0175	2.148
10	0.05	45.0	0.0175	2.072

Our design optimization process using MGN remarkably accelerates the evaluation of design configurations compared to CFD simulations. By employing our MGN-based surrogate model, the design optimization procedure takes < 4 h, whereas the same optimization using CFD would have taken approximately 3,000 h. However, this acceleration comes with a tradeoff between accuracy and speed. While our surrogate model maintains an average relative error in IA predictions of 9.2%, it is sensitive to certain parameter configurations. These discrepancies highlight the importance of balancing computational efficiency with the need for precision in critical regions of the design space. Despite these challenges, the accelerated design optimization process remains highly promising for practical applications, allowing for rapid improvement of carbon capture system designs.

## 4 Discussion

We reported an improved MGN-based surrogate model that accelerates the numerical simulations of fluid dynamics in a carbon capture column and operates in a large design space. Compared to classical CFD approaches with heavy computational cost, we showed that our data-driven MGN model can achieve over 500 × speedup on a single modern GPU and 1,700 × speedup with parallel computing and other enhancements. Despite having to learn the the dynamics across a larger design space than in our previous works, our model still successfully predicts the fluid dynamics and achieves low prediction error in IA across wide ranges of liquid inlet velocity, contact angle, and surface area inputs, with under 10% average test relative error in IA. The high inference speed and low error of this model holds the potential for fast and accurate exploration of large parameters for design optimization, and we showed that that our model can be used to rapidly explore our design space to optimize IA.

Even with improved acceleration and low error of our model, the MGN-based approach can still be improved for larger-scale applications. We observed that RMSE_VF_ and IA error calculated from the model predictions were not always consistent with each other. IA error was sensitive to the discrepancies in the predicted and true interfaces near the packing structure, while RMSE_VF_ reflected the training loss for global volume fraction prediction. Thus, training and optimizing MGN based on a loss function that is indifferent to the packing structure is not necessarily optimal for minimizing IA error. Future work may consider adaptively putting more training error weight around packing structure regions most relevant to the IA computation for improved results.

Another aspect to consider is that this model was trained using limited computational resources and can be further improved for better accuracy by increasing size of the model or a more extensive hyperparameter search. Specifically, we performed ablation studies that confirmed that increasing number of message-passing steps and hidden dimension size in MGN can improve model capacity (Supplementary Figures S6, S7). Advanced model architectures can also be explored for improvements, including multi-scale learning (Lino et al., 2021; Fortunato et al., 2022), multi-step learning with longer history (Han et al., 2022), physics-guided approaches (Hu et al., 2023), graph neural operators (Li et al., 2020), and differentiable design (Allen et al., 2022). Introducing these cutting-edge enhancements can not only benefit the 2D modeling of the carbon capture column, but also enables the application to 3D modeling, which involves extremely higher complexity and resource requirements (Bartoldson et al., 2023).

In conclusion, this study marks a crucial step toward rapid design optimization for carbon capture systems. By establishing accurate surrogate models that significantly accelerate CFD simulations, this research opens up the possibilities for more rapid progress and optimization in the development of carbon capture technologies.

## Data Availability

The datasets presented in this study can be found in online repositories. The names of the repository/repositories and accession number(s) can be found below: https://data.pnnl.gov/group/nodes/dataset/33472.
