# Supplementary material for: Accelerating computational fluid dynamics simulation of post-combustion carbon capture modeling with MeshGraphNets
Source: Front Artif Intell. 2025 Jan 7;7:1441985. doi: 10.3389/frai.2024.1441985 (PMC11752894; doi:10.3389/frai.2024.1441985)
Supplement: Supplementary file 1 [file Data_Sheet_1.pdf]

Supplementary Material

1 SUPPLEMENTARY INFORMATION

This section gives the formula for the computation of metric $\text{RMSE}_{\text{VF}-1}$, $\text{RMSE}_{\text{VF}-500}$ and the relative IA error. Denote G_t as the ground truth input graph at time t with $N = 164,715$ nodes. Denote $\mathbf{y}_t \in \mathbb{R}^N$ as the target volume fractions at time t, we train $\text{MGN}(G_t)$ to predict $\Delta \mathbf{y}_t = \mathbf{y}_{t+1} - \mathbf{y}_t$. We then predict the next state as $\hat{\mathbf{y}}_{t+1} = \mathbf{y}_t + \text{MGN}(G_t)$. The next-step error for a simulation with $T = 500$ steps is

$$\text{RMSE}_{\text{VF}-1} = \sqrt{\frac{\sum_{t=1}^{499} \|\mathbf{y}_{t+1} - \hat{\mathbf{y}}_{t+1}\|^2}{499N}} = \sqrt{\frac{\sum_{t=1}^{499} \|\Delta \mathbf{y}_t - \text{MGN}(G_t)\|^2}{499N}}.$$

By iteratively applying the MGN model and updating the graph with its predictions, we can obtain the full rollout and the last step prediction $\tilde{\mathbf{y}}_{500}$. The last-step error is

$$\text{RMSE}_{\text{VF}-500} = \sqrt{\frac{\|\mathbf{y}_{500} - \tilde{\mathbf{y}}_{500}\|^2}{N}}.$$

We compute steady-state IA as the average IA of the last 20 timesteps of a simulation. Denote IA_{gt} as the ground truth steady state IA and IA_{pred} as the predicted steady state IA. The relative IA error is defined as

$$\text{relative IA error} = \frac{|\text{IA}_{gt} - \text{IA}_{pred}|}{\text{IA}_{gt}} \times 100\%.$$

2 SUPPLEMENTARY TABLES

Table S1. One-step error on volume fraction $RMSE_{VF-1}$, last-step error on volume fraction $RMSE_{VF-500}$ and relative IA error for each test simulation.

	σ (N/m)	θ (°)	$\mathbf{v}_{inlet}$ (m/s)	$RMSE_{VF-1}$	$RMSE_{VF-500}$	relative IA Error (%)
1	0.01	50	0.00326	0.022	0.251	24.1
2	0.01	50	0.0141	0.041	0.314	27.3
3	0.01	70	0.00326	0.024	0.258	2.0
4	0.01	70	0.00864	0.036	0.266	10.6
5	0.01	70	0.0218	0.053	0.334	10.5
6	0.01	90	0.0218	0.059	0.327	30.3
7	0.03	10	0.0141	0.038	0.276	3.3
8	0.03	30	0.0218	0.047	0.325	2.1
9	0.03	50	0.00531	0.025	0.305	7.1
10	0.03	70	0.0218	0.051	0.350	4.0
11	0.03	90	0.00864	0.037	0.302	6.3
12	0.03	90	0.0141	0.045	0.326	4.6
13	0.05	10	0.002	0.012	0.245	5.0
14	0.05	30	0.002	0.013	0.290	30.1
15	0.05	30	0.00326	0.017	0.296	10.6
16	0.05	50	0.002	0.013	0.328	20.0
17	0.05	50	0.00326	0.018	0.282	11.8
18	0.05	90	0.00531	0.027	0.296	5.0
19	0.07	10	0.00531	0.021	0.302	6.1
20	0.07	30	0.00864	0.028	0.315	2.2
21	0.07	30	0.0218	0.042	0.342	5.1
22	0.07	70	0.00531	0.026	0.377	5.6
23	0.07	90	0.002	0.017	0.289	8.8
24	0.07	90	0.0141	0.041	0.369	12.6
25	0.09	10	0.002	0.014	0.308	2.9
26	0.09	10	0.00864	0.029	0.338	3.9
27	0.09	10	0.0141	0.035	0.368	1.2
28	0.09	30	0.00326	0.018	0.332	1.0
29	0.09	50	0.00864	0.031	0.344	6.5
30	0.09	70	0.00531	0.027	0.350	4.0
Avg.	-	-	-	0.030	0.313	9.2

3 SUPPLEMENTARY FIGURES

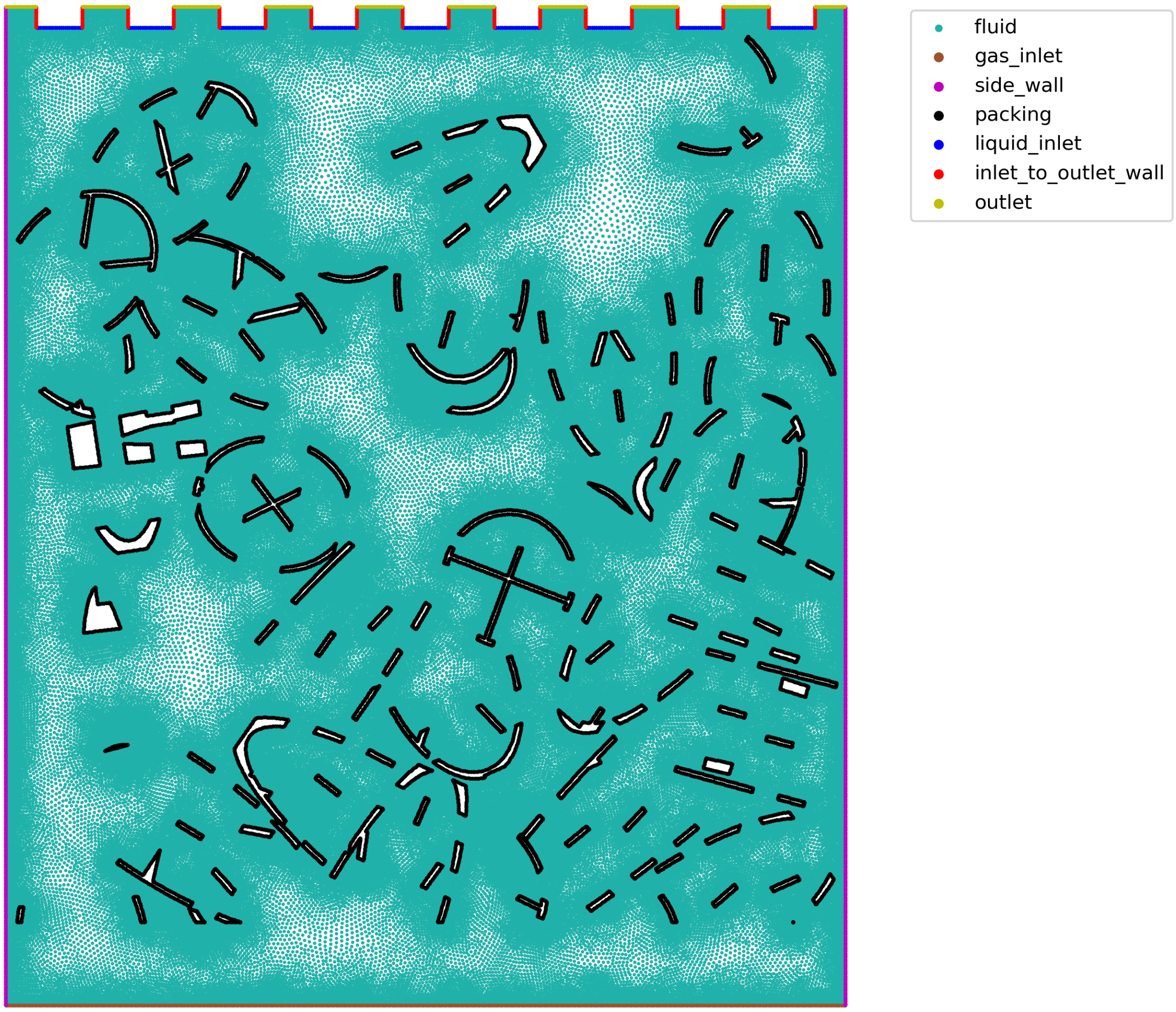

Figure S1. Node types for 2D mesh. Node types are fluid, gas_inlet (inlet nodes for CO_2 at the bottom), side_wall, packing (surface of packing structure), liquid_inlet (inlet nodes for liquid solvent), inlet_to_outlet_wall and outlet (outlet nodes for CO_2 on the top).

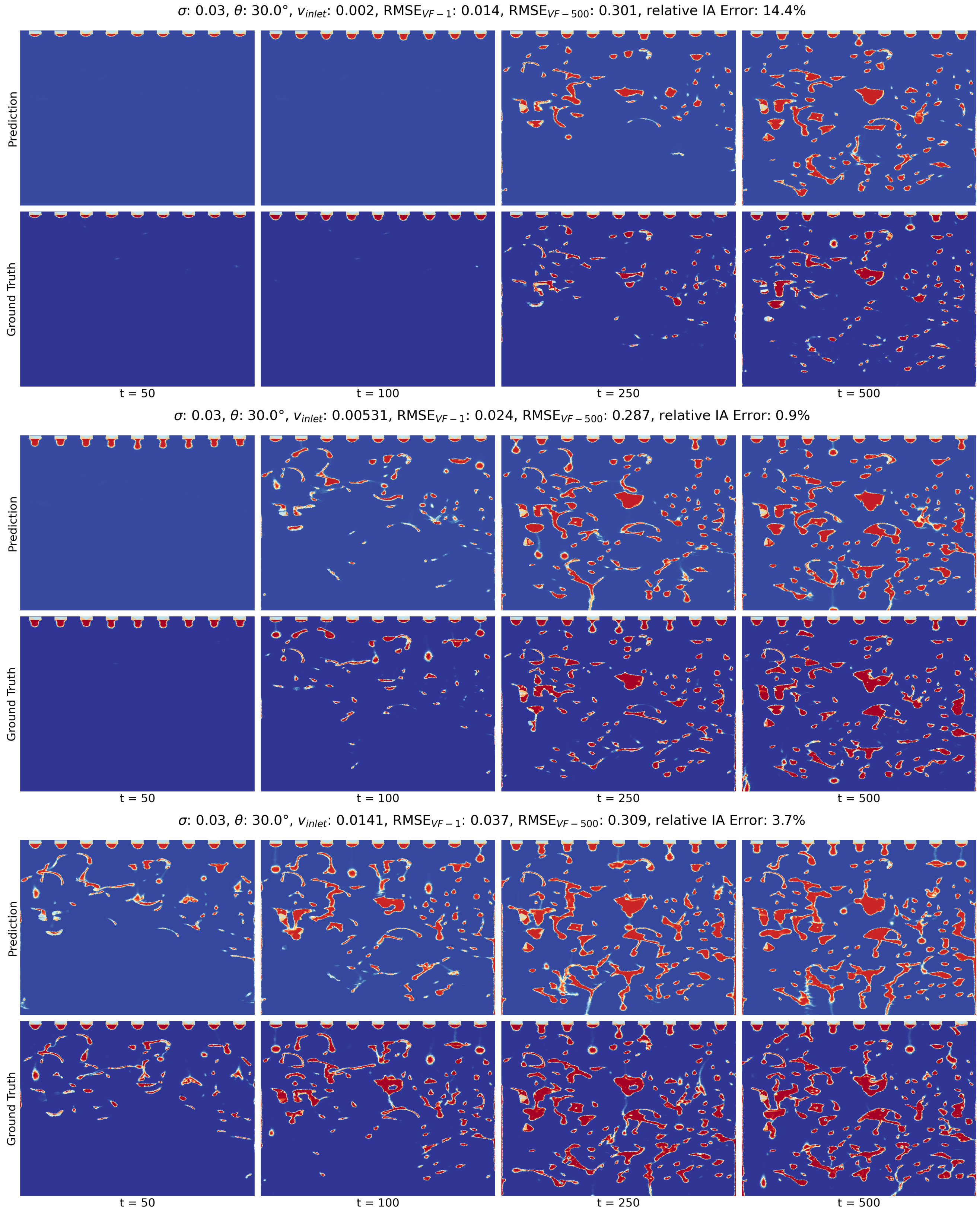

Figure S2. Predicted and CFD-generated rollouts of the volume fraction for 3 train simulations with increasing $\mathbf{v}_{\text{inlet}}$ and fixed $\theta = 30°$ and $\sigma = 0.03$. In each subplot, the top and bottom rows correspond to the predicted and ground truth rollouts, respectively. From left to right, the time steps are 50, 100, 250 and 500.

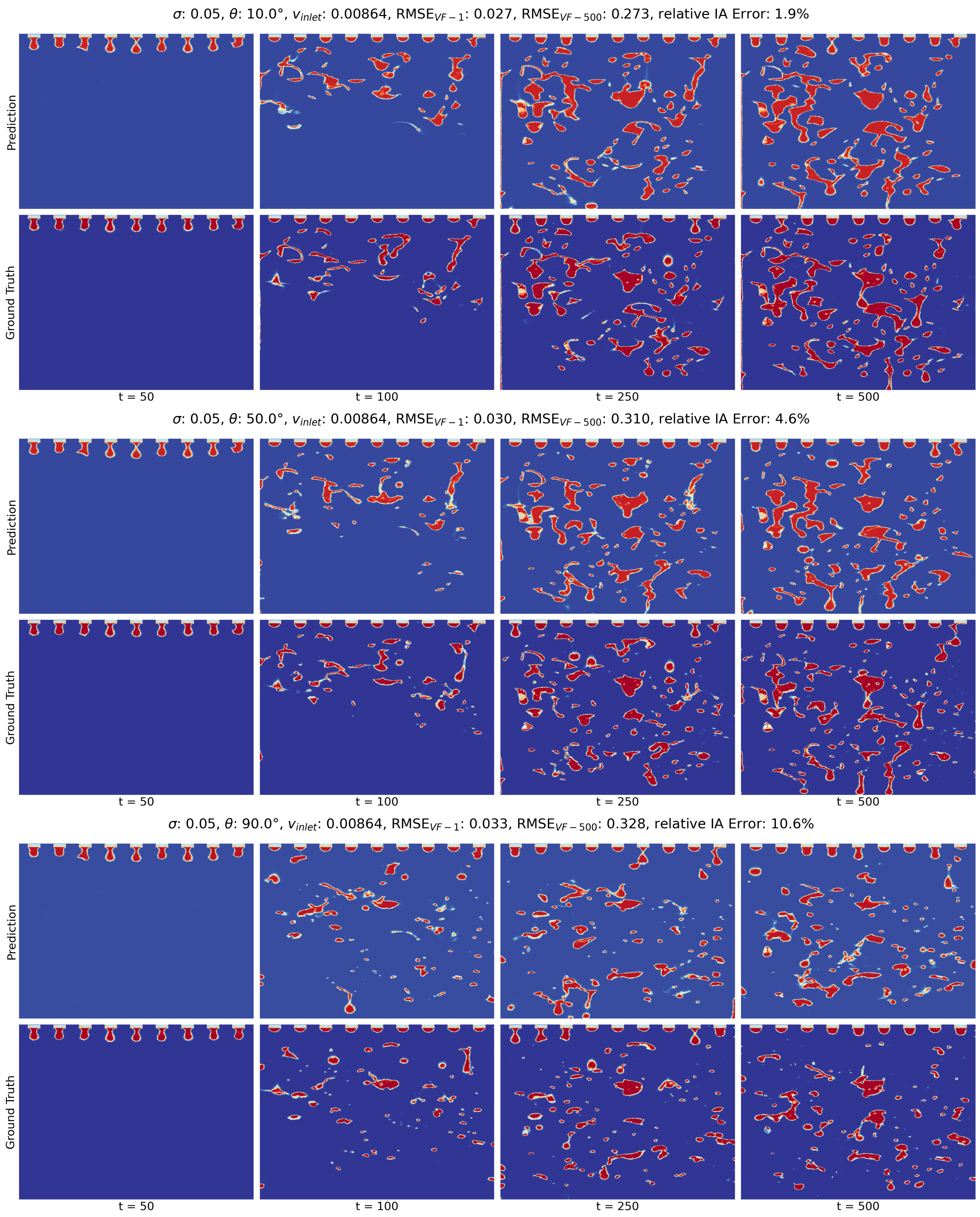

Figure S3. Predicted and CFD-generated rollouts of the volume fraction for 3 train simulations with increasing θ and fixed $\sigma = 0.05$ and $\mathbf{v}_{\text{inlet}} = 0.00864$. In each subplot, the top and bottom rows correspond to the predicted and ground truth rollouts, respectively. From left to right, the time steps are 50, 100, 250 and 500.

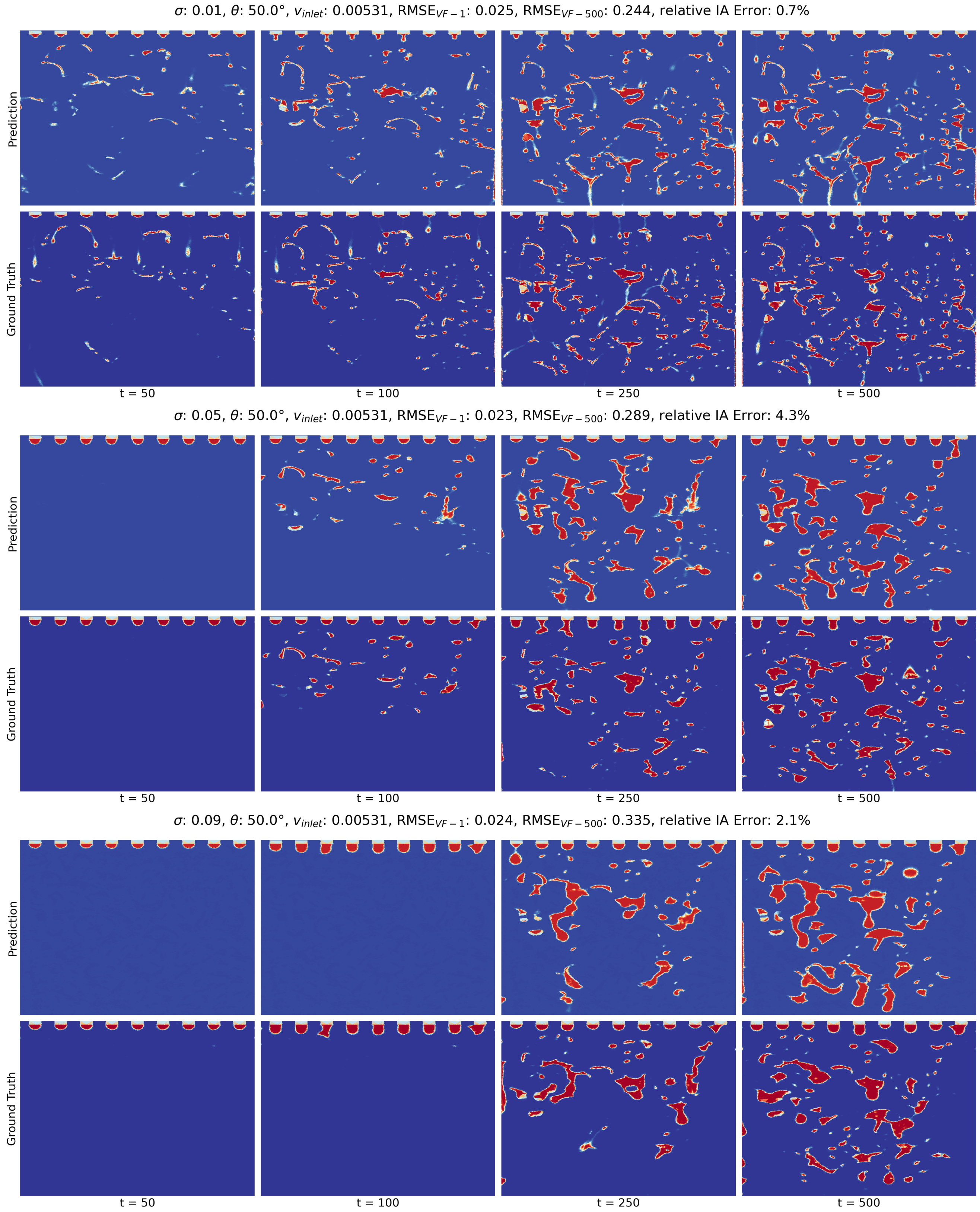

Figure S4. Predicted and CFD-generated rollouts of the volume fraction for 3 train simulations with increasing σ and fixed $\theta = 50°$ and $\mathrm{v}_{\mathrm{inlet}} = 0.00531$. In each subplot, the top and bottom rows correspond to the predicted and ground truth rollouts, respectively. From left to right, the time steps are 50, 100, 250 and 500.

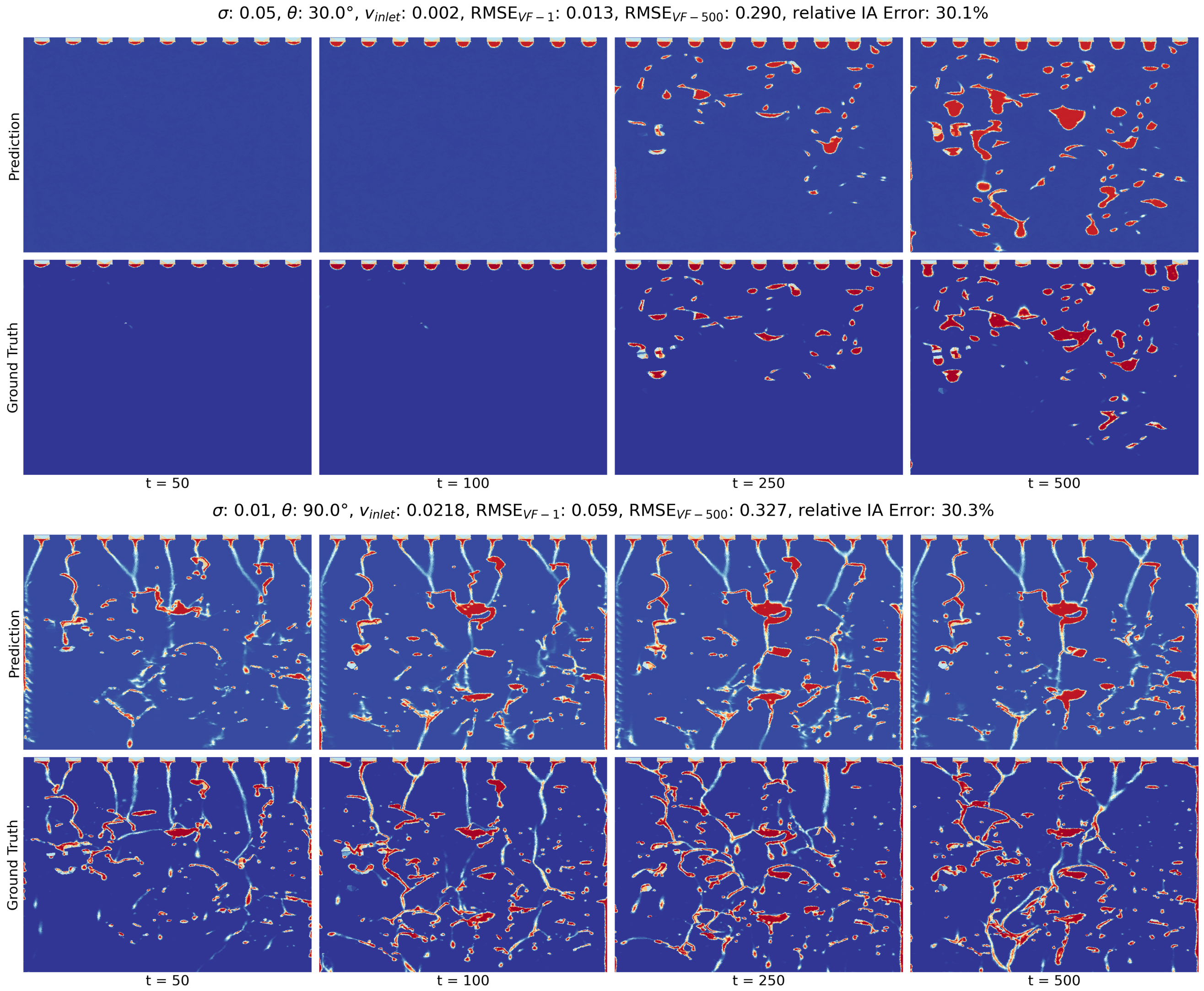

Figure S5. Predicted and CFD-generated rollouts of the volume fraction for two simulations exhibiting suboptimal results in IA prediction. The first case features a lowest $\mathbf{v}_{\text{inlet}}$ at 0.002 and the second case features a highest $\mathbf{v}_{\text{inlet}}$ at 0.0218.

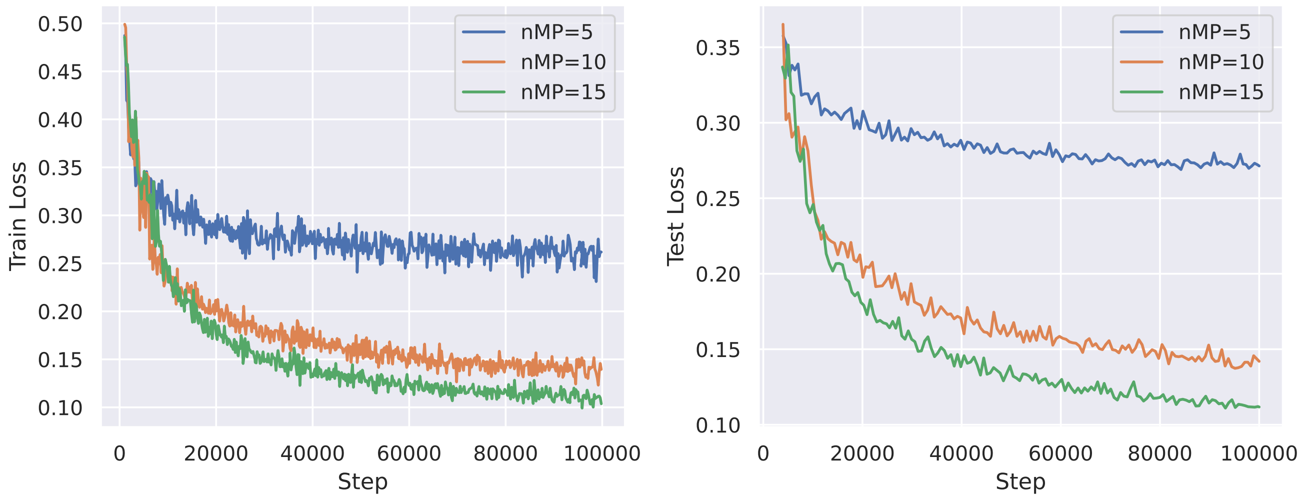

Figure S6. Train and test learning curves for 5, 10, and 15 message-passing steps. Models are trained for 100k steps. Increasing the number of message-passing steps significantly improves learning.

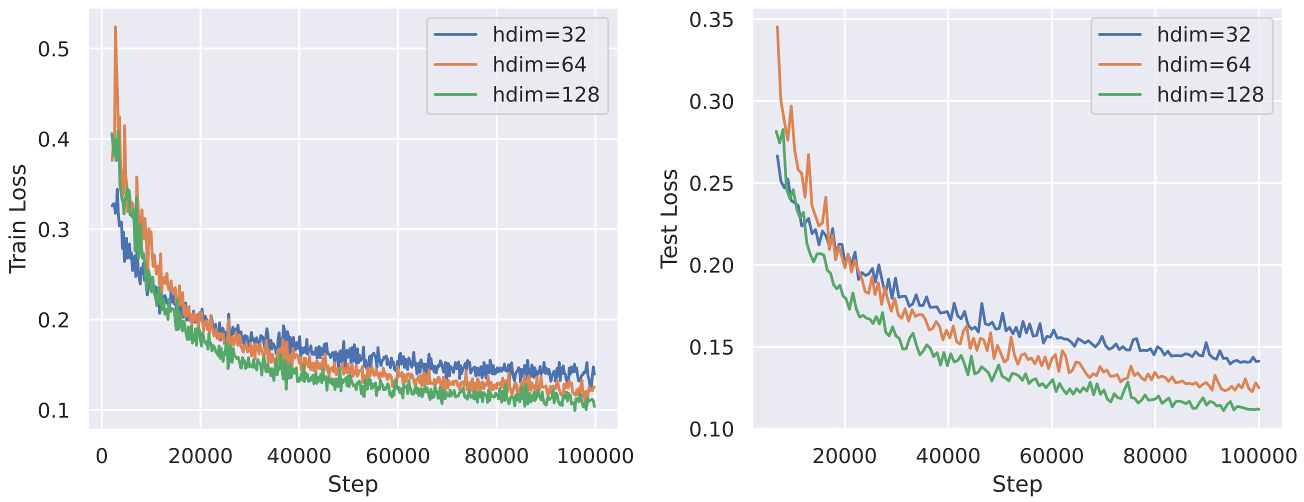

Figure S7. Train and test learning curve for models with hidden dimensions of 32, 64 and 128. Models are trained for 100k steps. Increasing the size of the hidden dimension results in moderate improvements in learning. However, the number of hidden dimensions has a less significant impact on performance compared to the number of message-passing steps.

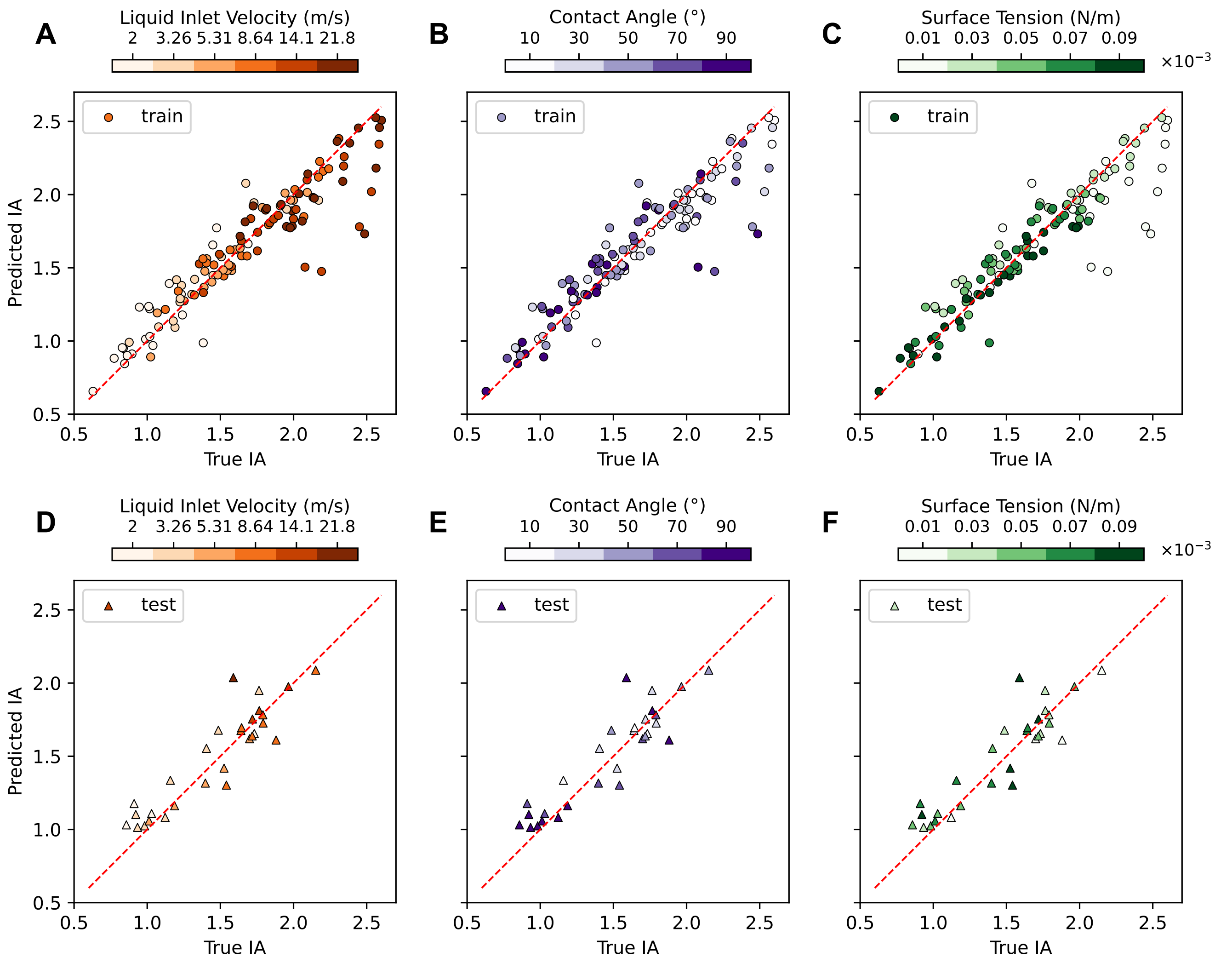

Figure S8. Predicted vs. true IA for the train (1st row) and test (2nd row) simulations. The same predictions are shown in each panel but are colored by (A,D) liquid inlet velocity, (B,E) contact angle, and (C,F) surface tension of the corresponding simulation. The relative IA error for the train and test sets is 7.6% and 9.2%, respectively.

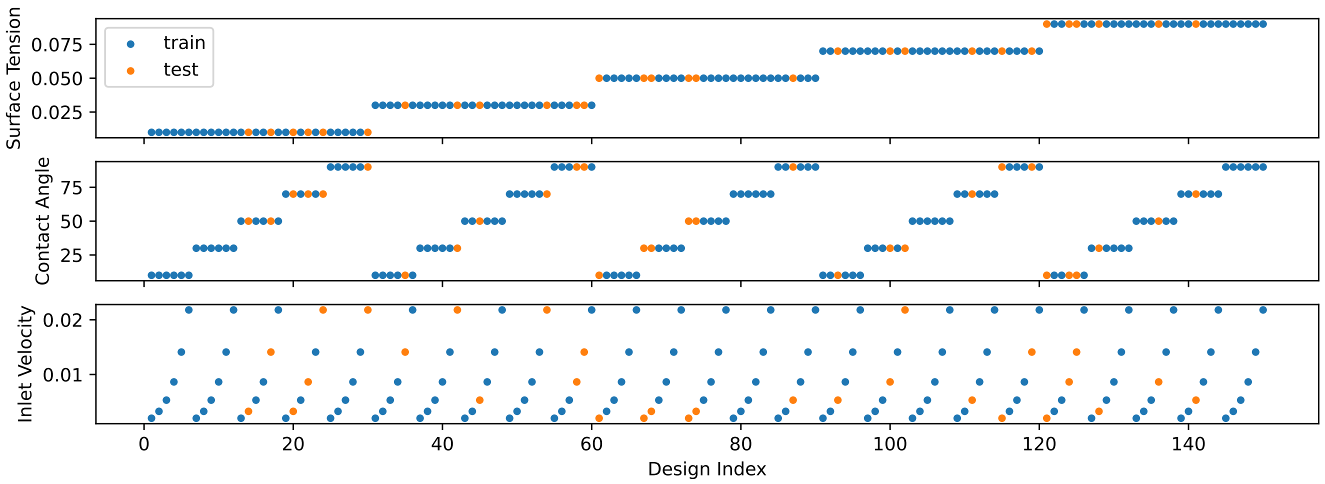

Figure S9. The training and test dataset split obtained using Latin hypercube sampling over 150 simulations in total.

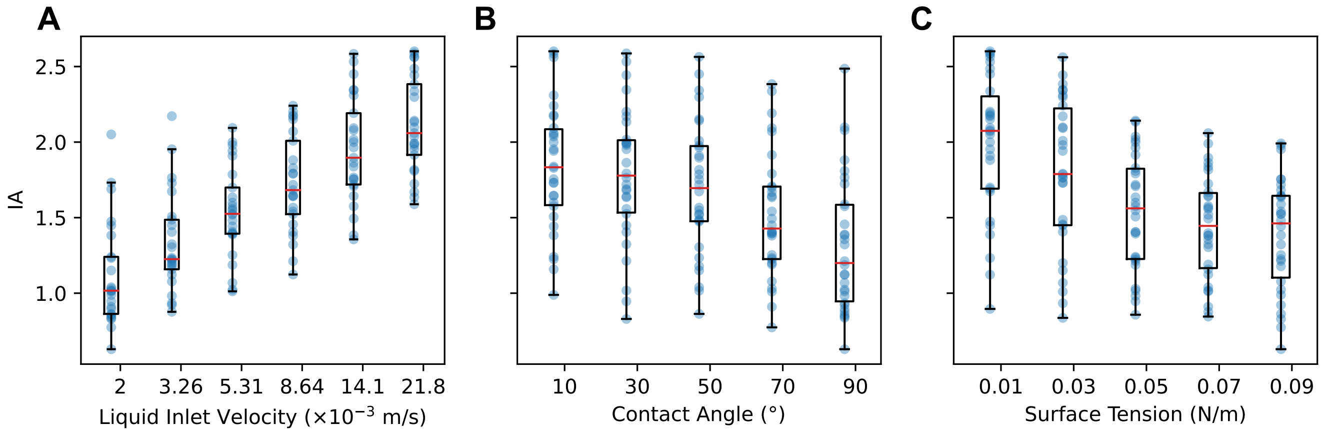

Figure S10. True interfacial area (IA) vs. (A) liquid inlet velocity, (B) contact angle, and (C) surface tension. The combination of these three design parameters has a large impact on the true IA value.

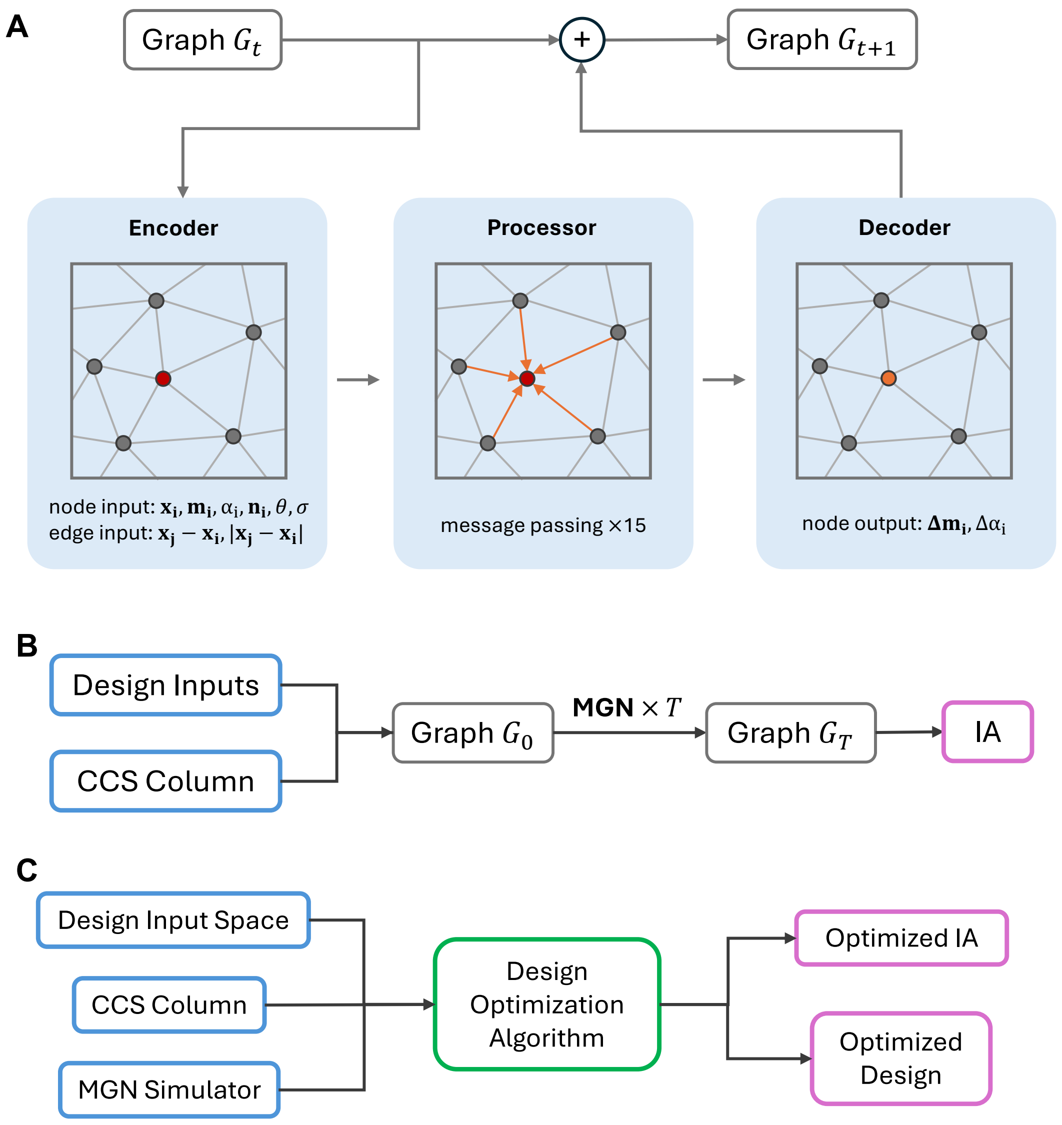

Figure S11. (A) Diagram of the MeshGraphNets model, which operates by a next-step prediction. The encoder transforms both node and edge inputs into latent features. Node inputs include physical location $\mathbf{x}_i$, momentum per unit volume $\mathbf{m}_i$, liquid volume fraction α_i, node type $\mathbf{n}_i$, contact angle θ and surface tension σ. Edge inputs consist of the relative displacement vector $\mathbf{x}_j - \mathbf{x}_i$ and the distance $|\mathbf{x}_j - \mathbf{x}_i|$. The processor updates the node and edge features with 15 message passing steps. The decoder obtains $\Delta\mathbf{m_i}$ and $\Delta\alpha_i$, which are used to update states from time step t to $t+1$. (B) Workflow of computing interfacial area (IA). The design inputs and CCS column mesh are used to build graph G_0. MGN is then iterately applied T times to get the final graph G_T, followed by computation of IA. (C) Workflow of design optimization. The design optimization algorithm takes the range of design inputs, CCS column and trained MGN simulator as inputs and output the optimized IA and optimized design parameters.
